# Global research trends in the relationship between diabetic cardiomyopathy and mitochondria: a bibliometric analysis

**DOI:** 10.1186/s41065-025-00488-3

**Published:** 2025-07-01

**Authors:** Jiajie Li, Jinxing Liu, Yaping Wang, Heguo Yan, Qin Li, Weibo Wen

**Affiliations:** 1 Yunnan Key Laboratory of Integrated Traditional Chinese and Western Medicine for Chronic Disease in Prevention and Treatment, Yunnan University of Chinese Medicine, No. 1076, Yuhua Road, Chenggong District, Kunming, 650500 China; 2 Joint Graduate School of Traditional Chinese Medicine of China, Suzhou, 215105 China; 3 School of Integrated Chinese and Western Medicine, Anhui University of Chinese Medicine, Hefei, 230012 China; 4 School of Basic Medical Sciences, Yunnan University of Chinese Medicine, Kunming, 650500 China

**Keywords:** Diabetic cardiomyopathy, Mitochondria, Bibliometric analysis, Citespace, VOSviewer

## Abstract

**Background:**

Diabetic Cardiomyopathy (DCM) is a distinct form of heart disease whose pathogenesis remains largely elusive. Recent studies have shed light on the significant role of mitochondria in the development of DCM, emphasizing their critical involvement. Despite these advancements, a bibliometric analysis focusing on the nexus between mitochondria and DCM has not been conducted, leaving a gap in a holistic understanding of research trends in this field.

**Methods:**

This study extracted publications addressing the role of mitochondria in DCM from the Web of Science Core Collection, spanning from 1988 to 2024. A detailed bibliometric analysis was undertaken using tools like CiteSpace, VOSviewer, Microsoft Excel, and Tableau Public to assess the data.

**Results:**

The analysis encompassed 440 publications involving 2705 researchers from 1457 institutions across 175 countries/regions. These studies were disseminated across 202 journals. China was the most prolific country with 192 publications, followed by the United States with 156, and Canada with 26. E. Dale Abel emerged as the most prolific author in this area. Key journals contributing to this research included the American Journal of Physiology-Heart and Circulatory Physiology and the Journal of Molecular and Cellular Cardiology. The future research direction is likely to focus deeper into the mechanisms of mitochondrial dysfunction in the diabetic heart and to identify molecular and cellular targets for therapeutic intervention.

**Conclusion:**

This report presents the first detailed bibliometric review of the intersection between mitochondrial research and DCM. It offers critical insights and guidance for researchers aiming to navigate and contribute to this evolving area of study.

## Introduction

Diabetic cardiomyopathy (DCM), a significant vascular complication of diabetes mellitus, is characterized by structural and functional myocardial changes stemming from metabolic disturbances [1]. These changes begin as subclinical alterations in cardiac function and can progress to heart failure. The prevalence of DCM among diabetic patients is reported to be between 30% and 60%. With the global rise in diabetes and its cardiovascular complications, diabetic cardiovascular disease has emerged as the primary cause of mortality among diabetic patients [2, 3]. Despite a significant increase in the number of studies targeting DCM over the past decade [4], there remains a lack of consensus on effective strategies for the prevention or treatment of diabetes-related cardiovascular disease. Mitochondria, the primary energy generators for cardiomyocytes, are crucial in supporting cardiac function [5]. It has been found that abnormalities and dysfunction in mitochondrial morphology are significant contributors to the development of DCM [6, 7]. Recent advancements in technology, including small molecule drugs that adjust mitochondrial mass, nanomolecular materials that target mitochondria precisely, and innovative cellular therapies, have shown promising potential in treating severe mitochondrial-associated diseases [8–11]. These findings suggest that mitochondrial-targeted therapies might represent a new avenue for DCM intervention [12].

Currently, researchers are continuously exploring the mechanisms behind mitochondrial defects. Recent advancements in understanding mitochondrial biology have unveiled numerous mitochondria-related pathways and mechanisms. These discoveries could offer crucial insights into the development of diabetic cardiomyopathy [13–15]. Despite these advancements, there remains a lack of bibliometric studies focusing specifically on mitochondrial research within diabetic cardiomyopathy. In this study, we utilized analytical tools such as CiteSpace and VOSviewer to perform statistical analyses and visualizations of the relevant literature, authors, institutions, and geographic distributions. Our goal was to map out the landscape of mitochondrial research related to diabetic cardiomyopathy over the last thirty years and pinpoint emerging trends in the field. This analysis aims to provide valuable insights that could encourage further research into this vital area [16].

## Methods

### Literature sources and search strategies

**Fig. 1:**
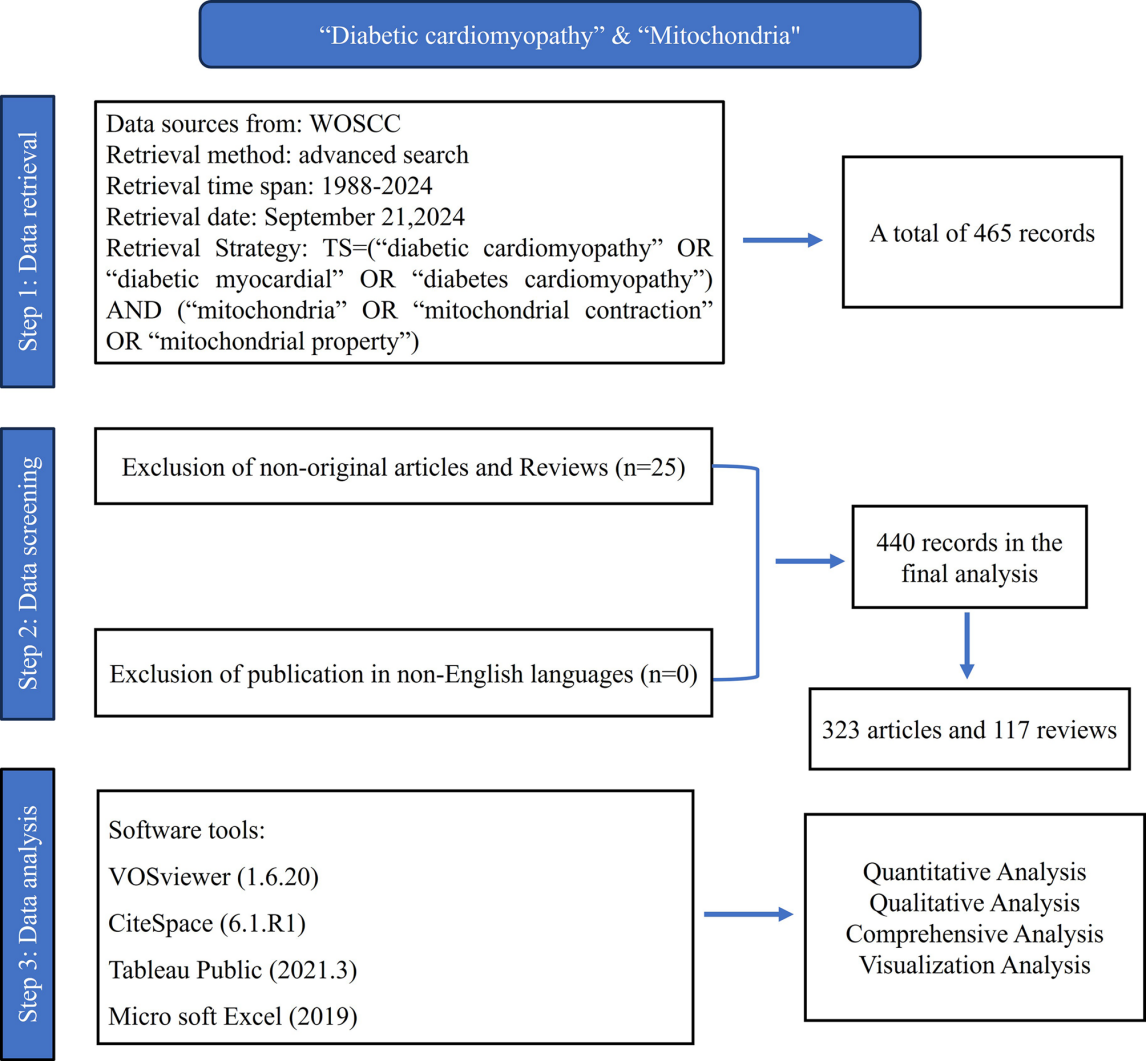
Flowchart depicting criteria for inclusion and exclusion of studies

To obtain research data, we conducted a literature search using the Web of Science Core Collection (WoSCC) database. We finalized our search on September 21, 2024, using the search terms TS= ((“diabetic cardiomyopathy” OR “diabetic myocardial” OR “diabetes cardiomyopathy”) AND (“mitochondria” OR “mitochondrial contraction” OR “mitochondrial property”)), covering publications from January 1, 1988, to September 21, 2024. This search yielded 465 records, which were then rigorously reviewed by two team members based on pre-determined inclusion and exclusion criteria, as depicted in Fig. 1. After this detailed screening process, 440 relevant articles were selected for inclusion. The essential details such as titles, authors, keywords, citations, journals, institutions, and references of these 440 articles were recorded and formatted in plain text.

### Analysis methods

In this study, we used various software tools for visualization and analysis, including CiteSpace 6.1.R1, VOSviewer 1.6.20, Microsoft Excel 2019, and Tableau Public 2021.3. Specifically, we employed CiteSpace and VOSviewer together to examine details of the publications such as country/region of origin, journal, author, references, and keywords, and each aspect was visualized separately. Additionally, we created a global distribution map of the publications using Tableau Public. A trend line depicting the annual publication volume was also generated using Microsoft Excel to provide a quantitative visualization of the data.

## Results

### Annual publication trends

**Fig. 2:**
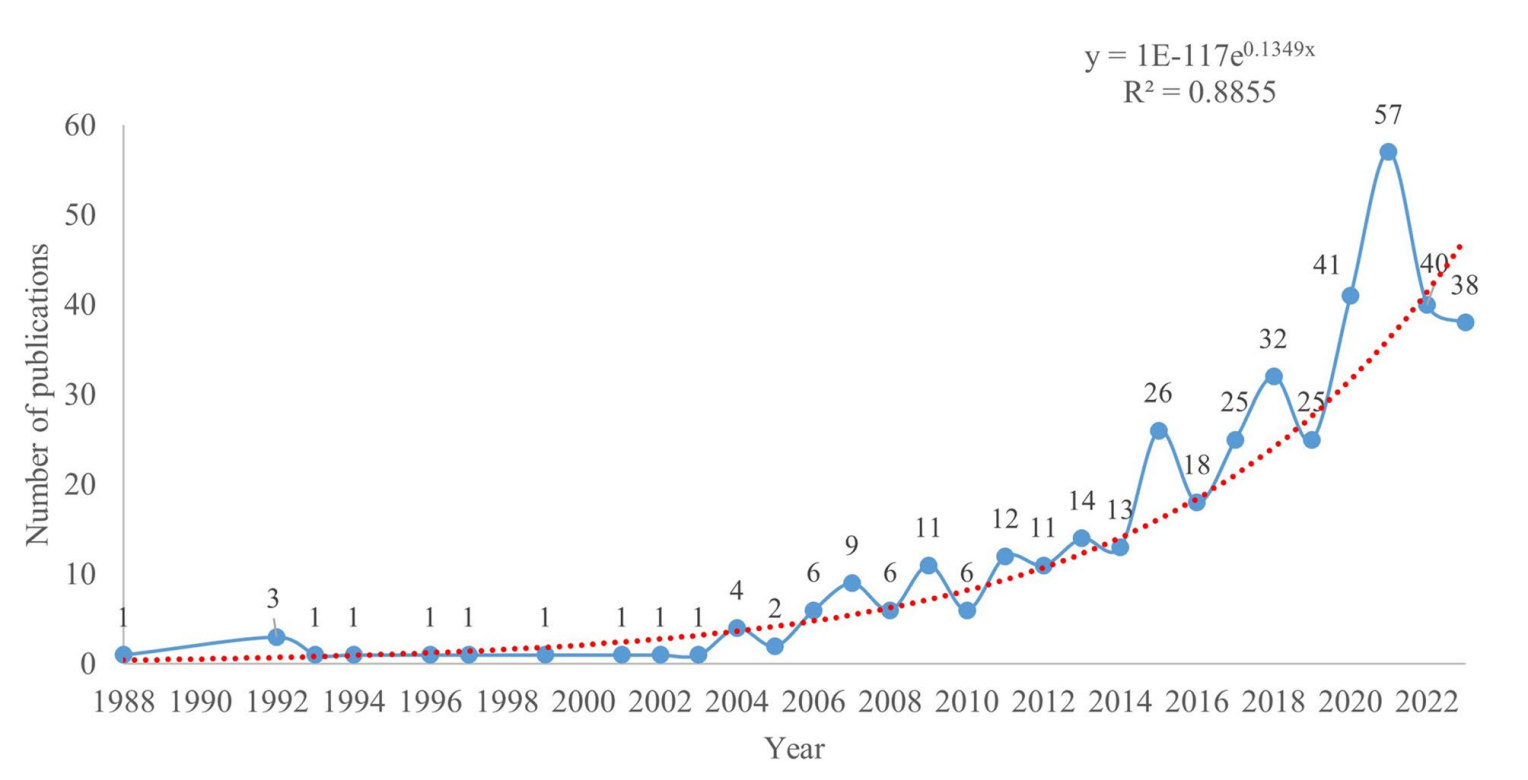
Publication trends in mitochondria-related DCM research

This analysis encompassed 440 publications concerning mitochondria in diabetic cardiomyopathy (DCM), comprising 323 research articles and 117 review papers. These works were authored by 2,705 researchers from 1,457 institutions across 175 countries/regions and were published in 202 different journals. The publication trends show a period of relative stability from 1988 to 2005, followed by a significant increase from 2006 to 2023, with the highest number of publications occurring in 2021 (*n* = 57) as illustrated in Fig. 2. Despite yearly fluctuations, a polynomial regression (R² = 0.8855) indicates a positive correlation between the year and the annual number of publications [17, 18], suggesting a growing interest in the intersection of DCM and mitochondrial research over the last three decades.

### Country and institutional analysis

**Fig. 3:**
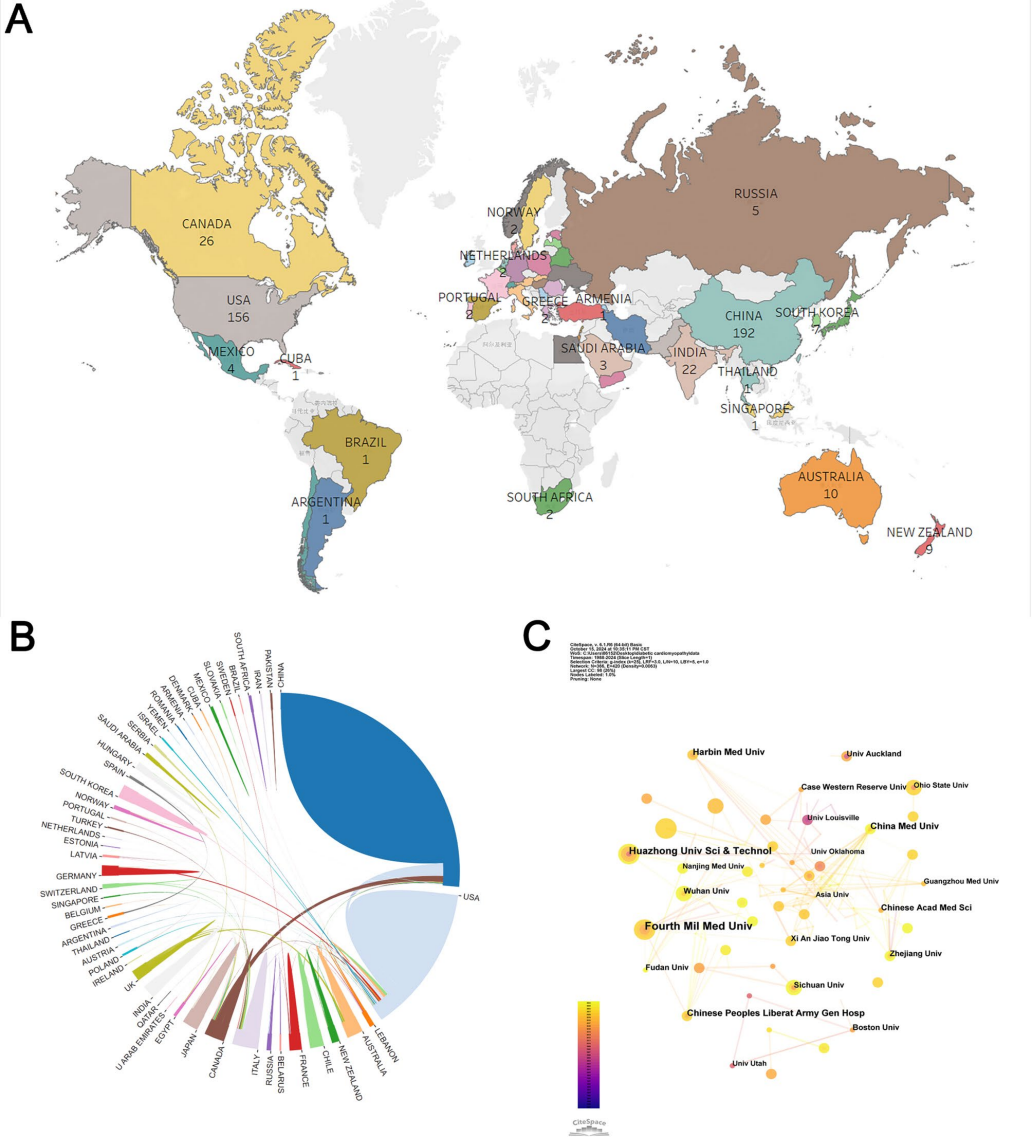
(**A**) Geographic Distribution Map: Displays the total number of publications by each country/region. (**B**) International Publication Collaboration Map: Illustrates the cooperative relationships in publication between different countries/regions. (**C**) Network of Institutional Collaborations: Indicates the connections and collaborative efforts among institutions focusing on research in DCM and mitochondria

175 countries/regions contributed to DCM and mitochondrial research. The global geographic distribution of this research, based on total publication numbers from each country/region, is illustrated in Fig. 3A. Table 1 identifies the top 10 countries and institutions leading in publication output. China is at the forefront with 192 publications, followed by the United States with 156, Canada with 26, India with 22, and Italy with 20. Notably, the majority of prolific publishing countries are located in Asia, North America, and Europe.

**Table 1:** Top 10 countries/regions and institutions by number of publications

Rank	Country	Records	Institutions	Records
1	CHINA	192	Air Force Medical University	15
2	USA	156	Huazhong University of Science and Technology	11
3	CANADA	26	China Medical University	8
4	INDIA	22	Chinese People’s Liberation Army General Hospital	8
5	ITALY	20	Harbin Medical University	8
6	JAPAN	14	Chinese Academy of Medical Sciences	7
7	ENGLAND	12	Boston University	6
8	GERMANY	12	University of Auckland	6
9	AUSTRALIA	10	Fudan University	6
10	NEW ZEALAND	9	Zhejiang University	6

In terms of international collaborations, there is a clear pattern where the extent of cooperation correlates positively with publication output, as shown in Fig. 3B. China, holding the highest publication count, has notably strong collaborations with the United States and Canada. Similarly, the United States has formed the broadest international partnerships in this research area.

Figure 3C primarily illustrates the collaborative networks among institutions. Table 1 highlights that Air Force Medical University (formerly The Fourth Military Medical University) in China leads with 15 publications, followed by Huazhong University of Science and Technology with 11, and China Medical University, Chinese People’s Liberation Army General Hospital, and Harbin Medical University each with 8 publications. The collaboration map in Fig. 3C reveals that Air Force Medical University, Chinese People’s Liberation Army General Hospital, Harbin Medical University, China Medical University have more collaborations with other institutions.

### Journal analysis

A total of 202 journals have published studies related to mitochondria in DCM. Among them, American Journal of Physiology-Heart and Circulatory Physiology has published 16 articles, followed by the Journal of Molecular and Cellular Cardiology with 15 articles. The International Journal of Molecular Sciences contributed 13 articles, Frontiers in Physiology published 12 articles, and Oxidative Medicine and Cellular Longevity produced 11 articles (Table 2).

**Table 2:** Top 10 journals and most frequently co-cited journals in mitochondria-related DCM research

Rank	Journals	Counts	IF (2024)	Co-Cited Journals	Counts	IF (2024)
1	American journal of physiology-heart and circulatory physiology	16	4.1	Circulation research	1390	16.5
2	Journal of molecular and cellular cardiology	15	4.9	Diabetes	1330	6.2
3	International journal of molecular sciences	13	4.9	Journal of biological chemistry	1257	4
4	Frontiers in physiology	12	3.2	Circulation	1151	35.5
5	Oxidative medicine and cellular longevity	11	—	American journal of physiology-heart and circulatory physiology	885	4.1
6	Diabetes	10	6.2	Journal of molecular and cellular cardiology	787	4.9
7	Free radical biology and medicine	10	7.1	Proceedings of the national academy of sciences of the United States of America	704	9.4
8	Frontiers in cardiovascular medicine	10	2.8	Cardiovascular research	725	10.2
9	Antioxidants & redox signaling	9	5.9	Journal of clinical investigation	517	13.3
10	Biochimica et biophysica acta-molecular basis of disease	8	4.2	Nature	485	50.5

**Fig. 4:**
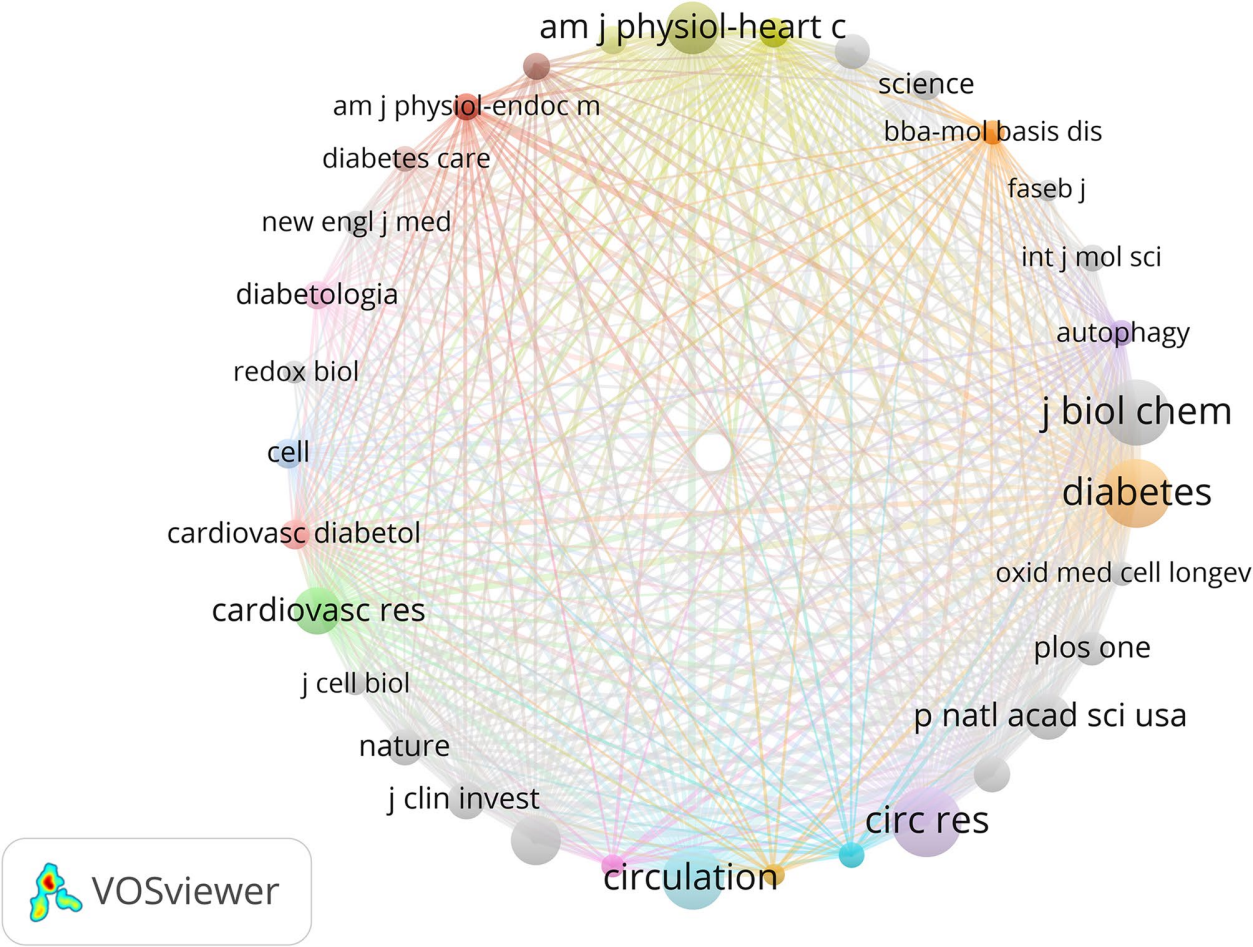
Network diagram of co-cited Journals in mitochondrial research for DCM

In this study, we analyzed a total of 440 publications, which were cited by 2,473 journals. The network of co-citations among these journals is depicted in Fig. 4, and the top 10 co-cited journals are listed in Table 2. Circulation Research leads with 1,390 citations, closely followed by Diabetes with 1,330 citations, and the Journal of Biological Chemistry with 1,257 citations. Other prominent journals in the top ten include Circulation and Nature, with 1,151 and 485 citations respectively. Notably, five of the top ten co-cited journals in 2024 have an impact factor above 10, with Nature boasting the highest at 50.5.

### Author analysis

**Fig. 5:**
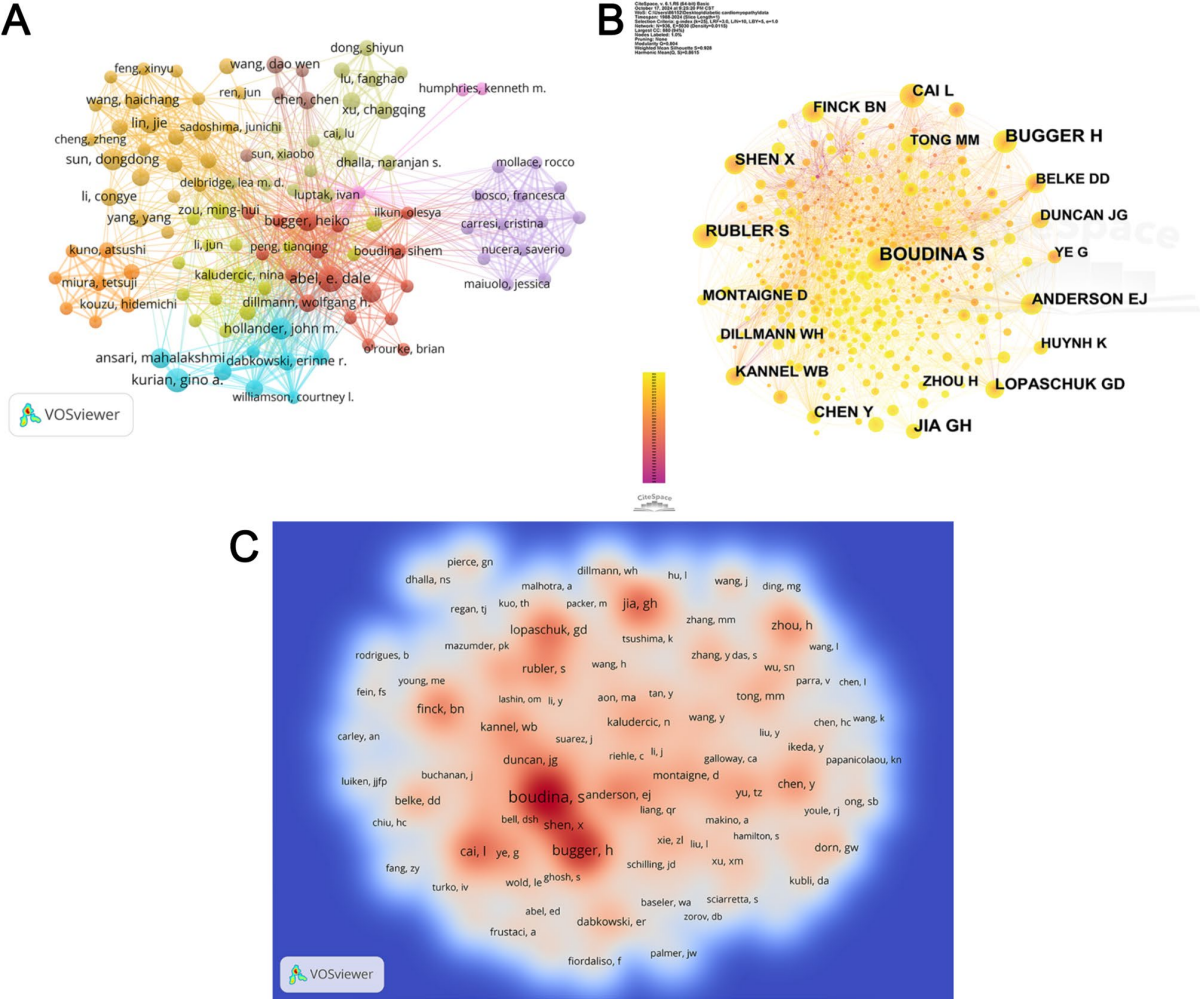
(**A**) Visualization network of author collaborations on mitochondrial research in DCM. (**B**) Visualization network of the various co-cited authors. (**C**) Density plot showing the distribution of citations among authors

A total of 2705 researchers have contributed to DCM and mitochondrial research over the past three decades. Figure 5A visually represents the collaborative relationships among these researchers. Table 3 lists the top 10 researchers based on their publication count, with Prof. E. Dale Abel from the Department of Medicine at the David Geffen School of Medicine at UCLA, USA, leading with nine publications in the field. Prof. Abel, who has held endowed professorships at the University of Utah and the University of Iowa, is celebrated for his groundbreaking work in metabolic regulation of the cardiovascular system, mitochondrial biology, and insulin signaling [19]. His laboratory has notably contributed to understanding how mitochondrial dysfunction and irregular insulin signaling heighten the risk of heart failure in diabetes [20].

**Table 3:** Top 10 most productive authors and co-cited authors in mitochondrial research related to DCM

Rank	Authors	Counts	Co-Cited Authors	Citations
1	Abel E Dale	9	Boudina Sihem	245
2	Kurian Gino A	8	Heiko Bugger	166
3	Hollander John M	7	Guanghong Jia	119
4	Dongdong, Sun	7	Lu Cai	107
5	Heiko Bugger	6	Lopaschuk Gary D	100
6	Ansari Mahalakshmi	6	Xia Shen	98
7	Wende Adam R	6	Finck Brian N	88
8	Dabkowski Erinne Rose	5	Hao Zhou	85
9	Croston Tara L	5	Anderson Ethan J	80
10	Qiangrong, Liang	5	Rubler S	74

The second highest number of publications was by Prof. Kurian Gino A from Vascular Biology Lab, School of Chemical and Biotechnology, SASTRA Deemed to be University, India. His research notably emphasizes mitochondrial mechanisms in the pathology of myocardial ischemia-reperfusion [21]. Prof. John M Hollander, positioned third, is from the Division of Exercise Physiology at the West Virginia University School of Medicine, USA. His lab is dedicated to developing novel therapies that target mitochondrial pathways to combat the bioenergetic deficits and impaired contractile functionality in diabetic heart failure patients, focusing particularly on the role of non-coding RNAs (miRNA, lncRNA, YRNA) and epigenetic changes in gene regulation [22]. Of note, Ansari Mahalakshmi also from Prof. Kurian’s lab, and Tara L Croston and Erinne Rose Dabkowski from Prof. Hollander’s team, are among the top ten authors, highlighting the significant research output from these labs.

Table 3 also features the top ten co-cited authors, with Sihem Boudina from the Department of Nutrition and Integrative Physiology at the College of Health, University of Utah, USA, leading with 245 citations. She is followed by Bugger Heiko with 166 citations, Jia Guanghong with 119 citations, Cai Lu with 107 citations, and Gary D Lopaschuk with 100 citations. Figure 5B displays the co-citation network among authors, while Fig. 5C underscores the influence of these co-cited authors in the domain of mitochondrial-related DCM research.

### Analysis of co-cited references and reference citation bursts

**Fig. 6:**
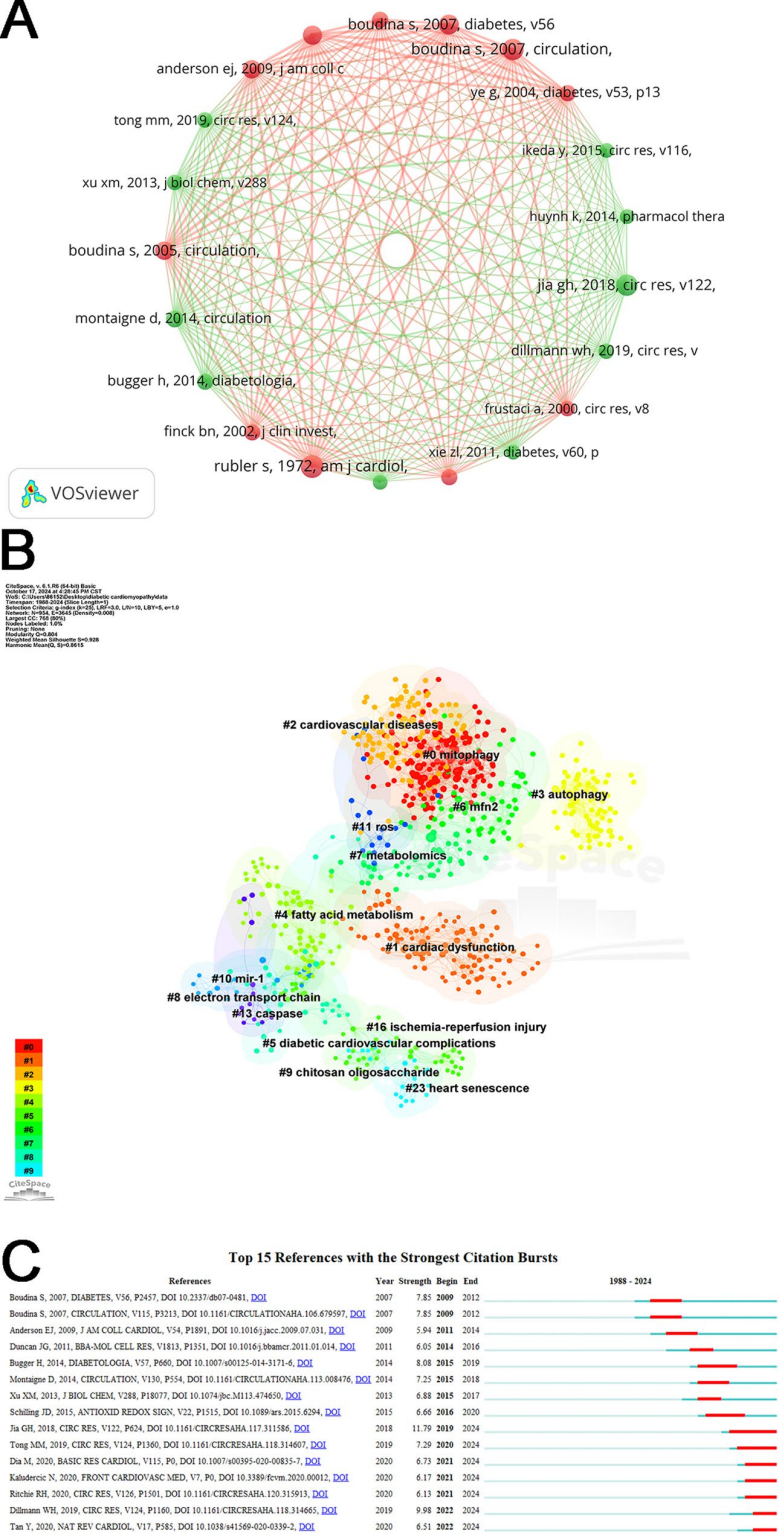
(**A**) Visualization network of co-cited references with at least 30 citations each. (**B**) Cluster analysis diagram of co-cited references. (**C**) List of the top 15 references experiencing the most significant citation bursts

A co-cited reference is a significant metric in scientific research, denoting a document cited simultaneously by two or more other publications. The frequency of citations a reference garners reflects its influence within the relevant field. Table 4 presents the top 10 co-cited references, detailing their citation counts and the nationalities of their corresponding authors. The most cited among these is an article by Rubler S et al. from 1972 in The American Journal of Cardiology, titled “New type of cardiomyopathy associated with diabetic glomerulosclerosis,” which accrued 73 citations. This pivotal study first confirmed the presence of diabetic cardiomyopathy through autopsies of four patients with diabetic glomerulosclerosis and heart failure, where diabetes was the sole identifiable cause of heart failure [23]. Of interest, 9 of the corresponding authors of the top 10 co-cited references were from the United States, reflecting the high acceptance of the United States in this research area. Figure 6A illustrates a visual network diagram of the co-cited references that received at least 30 citations. Using the LLR (log-likelihood ratio) clustering algorithm in CiteSpace, the keywords from these co-cited references were analyzed to identify prevalent themes. As shown in Fig. 6B, the highest-ranked cluster, labeled #0, pertains to mitophagy, underscoring its critical role in DCM research related to mitochondria. Clusters #1 and #2 correspond to cardiac dysfunction and cardiovascular diseases, respectively, indicating their prominence in the co-cited literature.

**Table 4:** Top 10 co-cited references in mitochondria-related DCM studies

Rank	Co-Cited Reference	Citations	Corresponding author’s country
1	Rubler S, 1972, AM J CARDIOL, V30, P595, DOI 10.1016/0002-9149(72)90595-4	73	USA
2	Boudina S, 2007, CIRCULATION, V115, P3213, DOI 10.1161/circulationaha.106.679597	67	USA
3	Jia GH, 2018, CIRC RES, V122, P624, DOI 10.1161/circresaha.117.311586	66	USA
4	Boudina S, 2007, DIABETES, V56, P2457, DOI 10.2337/DB07-0481	55	USA
5	Shen X, 2006, DIABETES, V55, P798, DOI 10.2337/Diabetes.55.03.06.DB05-1039	51	USA
6	Boudina S, 2005, CIRCULATION, V112, P2686, DOI 10.1161/circulationaha.105.554360	50	USA
7	Anderson EJ, 2009, J AM COLL CARDIOL, V54, P1891, DOI 10.1016/j.jacc.2009.07.031	49	USA
8	Montaigne D, 2014, CIRCULATION, V130, P554, DOI 10.1161/circulationaha.113.008476	42	France
9	Bugger H, 2014, DIABETOLOGIA, V57, P660, DOI 10.1007/S00125-014-3171-6	39	USA
10	Shen X, 2004, AM J PHYSIOL-ENDOC M, V287, PE896, DOI 10.1152/ajpendo.00047.2004	39	USA

Burst detection is used to identify abrupt increases in keyword or citation frequencies within a designated period, facilitating rapid analysis of emerging trends. Figure 6C illustrates the top 15 references that have demonstrated significant citation bursts. The first citation burst was observed in 2009, while the most recent occurred in 2022, with the longest burst lasting five years. The latest high-intensity burst was from a review by Wolfgang H Dillmann from the University of California, San Diego’s Department of Medicine, Division of Endocrinology/Metabolism. This review was published in Circulation Research in 2019, and it proposed a new vision of a possible therapeutic intervention for DCM by analyzing the molecular mechanisms of DCM mediated by cytoplasmic and mitochondrial calcium processing [24]. In addition, this review on DCM and mitochondrial dynamics continues to experience a surge in citations.

### Keywords analysis

**Fig. 7:**
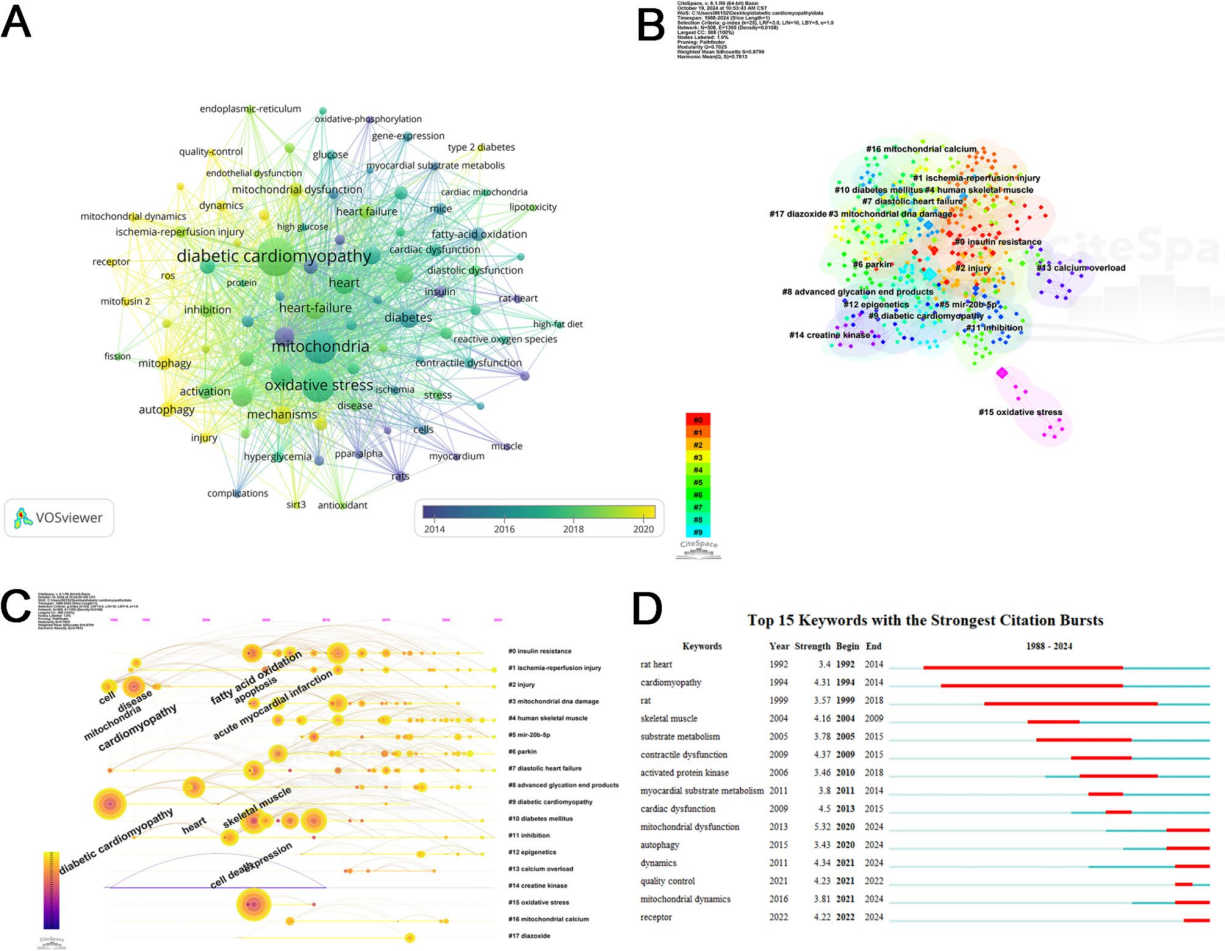
(**A**) Co-occurrence network diagram of keywords. (**B**) Graph showing clusters of keyword co-occurrences. (**C**) Timeline representation of keyword occurrences. (**D**) List of the top 15 keywords with the strongest citation bursts

Keywords succinctly represent the themes and content of an article, and their co-occurrence analysis not only deepens our understanding of the related research issues but also suggests directions for future studies. The network of keyword co-occurrences is depicted in Fig. 7A, while Table 5 lists the top 10 keywords by frequency. The terms “diabetic cardiomyopathy” and “mitochondria” lead the list with 259 and 182 occurrences, respectively. Notably, “oxidative stress” follows closely with 179 occurrences, highlighting the strong association between oxidative stress, mitochondria, and diabetic cardiomyopathy in current research. The frequency of these top three keywords significantly exceeded that of the others, underscoring their centrality in the field.

**Table 5:** Top 10 keywords in terms of frequency

Rank	Keywords	Counts	Rank	Keyword	counts
1	diabetic cardiomyopathy	259	6	heart-failure	78
2	mitochondria	182	7	apoptosis	73
3	oxidative stress	179	8	cardiomyopathy	67
4	dysfunction	84	9	diabetes	62
5	heart	80	10	mechanisms	57

The keyword information was further analyzed by cluster and timeline plots (Fig. 7B, C). Of interest in Fig. 7B, “mitochondrial DNA damage” emerged as the fourth most prominent theme among 18 clusters. In the context of diabetes, excessive glucose and metabolic anomalies prompt mitochondria to generate excessive reactive oxygen species (ROS), contributing to oxidative stress (OS). This increased OS is crucial for mitochondrial DNA (mtDNA) damage in cardiomyocytes, a key factor in the development of DCM [25]. Strategies to preserve mtDNA integrity and reduce mtDNA damage may slow DCM progression and offer new therapeutic targets [26]. In addition, Fig. 7C highlights the recent surge in research interest around mir-20b-5p in human microRNAs, marking it as an emerging and significant topic in the field.

Additionally, our analysis included the examination of keyword bursts. The top 15 keywords, identified by their citation bursts and displayed in Fig. 7D, are ranked by the onset of these bursts. It is evident that the focus areas in this field have evolved across three distinct phases: initially in the 1990s with keywords like “rat heart,” “cardiomyopathy,” and “rat”; then transitioning in the 21st century to “skeletal muscle,” “myocardial substrate metabolism,” and “cardiac dysfunction”; and more recently to “mitochondrial dysfunction,” “mitochondrial dynamics,” and “receptor.” This progression indicates a shift from early animal models of DCM to more complex studies of the molecular mechanisms involving mitochondria, positioning mitochondrial research as a key ongoing and future focus in this domain.

## Discussion

### Overview of mitochondrial research in the field of DCM

To investigate future research trends and hotspots, this study compiled and analyzed WoSCC data related to mitochondrial research in DCM. The analysis highlighted a steady yearly increase in publications. The data encompasses contributions from numerous countries, with China, the United States, Canada, India, and Italy being predominant. While China leads in the number of publications, the United States excels in international collaborations, thanks to its superior research infrastructure, ample funding, and interdisciplinary approach, attracting global researchers to its projects. Furthermore, the literature most frequently cited originates from the United States, emphasizing the significant international impact of American research. Considering this, it is recommended that China enhance its global research presence through increased international collaboration to foster innovation and greater influence in the field.

In terms of authors, Prof. E. Dale Abel of the Department of Medicine at the David Geffen School of Medicine at UCLA, USA, had the most publications with 9 papers in mitochondria-related DCM studies. In terms of journals, American Journal of Physiology-Heart and Circulatory Physiology leads in publication count in this domain. Among the top 10 co-cited journals in the field, seven have an impact factor greater than 5, and five exceed an impact factor of 10, underscoring the significant impact of research in this area.

### Knowledge base

A review of the highly cited literature provides a comprehensive understanding of the relationship between DCM and mitochondria. The development of DCM involves a complex cascade of events, and despite extensive research, its full pathogenesis remains elusive. Research indicates that the onset of DCM often begins with hyperglycemia triggered by insulin deficiency and resistance, initiating a cascade that impairs cardiac function [27, 28]. In the early stages of diabetes, the lack of insulin or insulin resistance induces cardiomyocytes to increase fatty acid uptake and β-oxidation to sustain ATP production. As the condition progresses, β-oxidation becomes insufficient for metabolizing fatty acids, leading to mitochondrial dysfunction. This dysfunction causes an accumulation of intracellular lipids and lipotoxicity [29], elevation of ROS, reactive nitrogen species (RNS), which further can drive oxidative and endoplasmic reticulum stress, suppress cellular autophagy, and ultimately lead to cardiomyocyte death, myocardial hypertrophy, and inflammation. Additionally, phosphorylation of myosin, which impacts myocardial passive tone and stiffness, contributes to cardiomyocyte hypertrophy and myocardial fibrosis. Disrupted Ca^2+^ cycling and increased fibrotic scarring also result in diabetic cardiac systolic dysfunction and arrhythmias, culminating in heart failure (HF) [30].

Mitochondrial dysfunction is recognized as a key pathological factor in the in the development of DCM [31]. Tuan H Kuo and colleagues were pioneers in documenting oxidative metabolism defects in the cardiac mitochondria of diabetic mice [32]. This discovery has led to numerous reports on changes in mitochondrial structure and function in DCM. Mitochondrial autophagy plays an essential role in regulating mitochondrial dynamics and mass. This process involves the selective removal of surplus, old, or damaged mitochondria through lysosomal degradation, which helps avert their harmful effects [33]. In turn, altered mitochondrial autophagy may be associated with cardiomyocyte death and the development of DCM [34]. Moreover, mitofusin 2 (Mfn2) is vital for mitochondrial fusion and maintaining mitochondrial and myocardial function. Mfn2 is a fusion protein located on the outer mitochondrial membrane that, when overexpressed, enhances mitochondrial fusion, aiding in the efficient functioning of mitochondria in cardiomyocytes. This action addresses the issue of excessive mitochondrial division in these cells [35]. Studies in recent years have shown that downregulation of Mfn2 is a key factor in mitochondrial dysfunction associated with DCM, accelerating its development. Conversely, Mfn2 overexpression can significantly mitigate the onset and progression of DCM [36].

In addition to changes in mitochondrial function, the therapeutic impact of drugs such as canagliflozin and melatonin on DCM has also been linked to improvements in mitochondrial dysfunction, highlighting it as a potential therapeutic target [37, 38]. Consequently, numerous studies have explored the modulation of mitochondrial function as a treatment strategy for DCM. For example, research has shown that mitochondrial aldehyde dehydrogenase can slow the progression of DCM in mice treated with streptozotocin, likely through mechanisms involving GSK3β, which helps maintain mitochondrial integrity, and Parkin, which promotes mitochondrial autophagy [39]. In addition, recent studies have shown that glutathione S-transferase P, interacts with Sirtuin 5 in mitochondria to undergo lysine desmalonylation, a process that helps prevent myocardial injury in DCM [40]. Various mechanisms by which mitochondria contribute to DCM include oxidative stress, basal metabolism, mitochondrial Ca^2+^ regulation, autophagy, dynamics, and biogenesis. Detailed studies are crucial to fully understand the potential pathogenesis of DCM and the specific roles mitochondria play in this disease process.

### New themes

An analysis of highly cited literature and keywords in this field highlights a focus on investigating new pathways that might contribute to mitochondrial dysfunction in hearts affected by diabetes. Exploration and discovery of targeting specific molecular and cellular pathways are important for diagnosis and prevention of DCM. Consequently, there is a growing emphasis on mitochondrial-targeted therapies as a novel approach in DCM treatment.

Mitochondrial dynamics, including fusion and fission, maintain the health of the mitochondrial network, which is essential for normal cell function. Mitochondria adapt to environmental changes by altering their shape, size, and number, processes involving fusion, fission, and autophagy [41]. In cases of DCM, factors such as insulin resistance and hyperglycemia can disrupt these dynamics, leading to the accumulation of damaged mitochondria. This results in mitochondrial fragmentation and dysfunction, decreasing ATP production and increasing ROS levels, which then contribute to cellular death and progression of the disease [42]. It has been found that modulating the expression of genes related to mitochondrial dynamics can be an effective therapeutic strategy for DCM. The protein Proliferator-activated receptor-γ coactivator-1α (PGC-1α), a key regulator of mitochondrial biogenesis, has been identified as a significant upstream factor in managing mitochondrial division and fusion [43]. Moreover, studies by Maayan Waldman and others have shown that sirtuin-1 (SIRT1) and its associated transcriptional coactivators, PGC-1α, and heme oxygenase-1 (HO-1), play significant roles in enhancing mitochondrial biogenesis and antioxidant defenses in DCM. Specifically, experiments involving a diabetic mouse model treated with angiotensin II showed that caloric restriction could modify cardiac remodeling through mechanisms linked to mitochondrial function and antioxidant pathways mediated by SIRT1 and PGC-1α [44, 45]. Additionally, increased HO-1 levels have been shown to bolster antioxidant defenses by interacting with SIRT1 and PGC-1α, thereby safeguarding diabetic hearts [46]. Although PGC-1α is known to regulate mitochondrial dynamics, enhance mitochondrial biogenesis, and reduce oxidative stress to mitigate DCM [47], recent studies have identified a contrasting role for PGC-1β. As a substrate of ubiquitin-specific protease 7, PGC-1β has been shown to contribute to metabolic disorders and mitochondrial dysfunction in the diabetic heart [48].

Excessive activation of Dynamin-related protein 1 (Drp1), a key player in mitochondrial dynamics, can contribute to cardiovascular diseases. In a hyperglycemic environment, the expression of Mfn1 in cardiomyocytes is reduced, whereas Drp1 expression is increased, promoting mitochondrial fission. This increased fission leads to higher ROS production and impaired insulin signaling, further exacerbating diabetic cardiomyopathy [49]. Research has shown that mitochondrial fragmentation in cardiomyocytes from diabetic mice can be restored by altering the phosphorylation level of Drp1 [50]. Notably, two serine residues on Drp1, located within its guanosine triphosphatase (GTPase) effector domain at the C-terminus, play contrasting roles in regulating mitochondrial fragmentation. Phosphorylation at the S616 site activates mitochondrial division, whereas phosphorylation at the S637 site suppresses it [51]. A recent study found that under conditions of high glucose and free fatty acids, silencing MAP4K4 can lead to decreased phosphorylation at the S616 site and increased phosphorylation at the S637 site., which in turn promoted the mitochondrial translocation of Drp1 to exacerbate mitochondrial fission, ultimately causing myocardial injury [52]. Additionally, the calcium channel protein Orai1 was also found to significantly influence Drp1 activity, facilitating mitochondrial fission through phosphorylation at the S616 site by ERK and dephosphorylation at the S637 site by calcium-regulated phosphatases. Intriguingly, Orai1 can activate both regulatory pathways [53].

Despite significant progress over recent decades in understanding the molecular underpinnings of DCM, many aspects remain unclear. Firstly, the intricate regulation of mitochondrial function involves multiple intracellular organelles, and the interplay between mitochondria and these organelles and their impact on DCM warrants further exploration; secondly, while current research primarily utilizes animal and cellular models, translating these findings into clinical applications is crucial for advancing treatment strategies [24]; finally, while moderate mitochondrial fusion, fission, and autophagy can protect the heart, excessive activity in these processes can disrupt mitochondrial function and contribute to DCM development. Thus, finding a balance in these mitochondrial processes is an urgent challenge that needs addressing. In conclusion, mitochondrial dysfunction continues to be a major factor in DCM pathogenesis, suggesting that future research should delve deeper into the multifaceted mechanisms causing mitochondrial dysfunction in the diabetic heart and focus on identifying specific molecular and cellular targets for therapeutic intervention. This approach should be a primary focus in the field moving forward.

## Strengths and limitations

Overall, utilizing tools like CiteSpace and VOSviewer enabled us to obtain a more detailed understanding of the key developments and trends in mitochondrial research in the field of DCM. However, this study has several limitations. Primarily, we restricted our search to English-language publications from the Web of Science Core Collection database, potentially overlooking pertinent research and impacting the completeness of our findings. Furthermore, there might have been an inadvertent introduction of selection bias during the process of literature screening.

## Conclusion

In this study, we conducted a bibliometric analysis on publications related to mitochondrial research within the context of DCM, marking the first comprehensive assessment in this field. The analysis revealed an annual increase in publications, indicating growing interest and research output in this area. The United States and China have emerged as key contributors, leading in both the quantity and impact of research on this topic. Among institutions, Air Force Military Medical University leads in publication volume. Among individual researchers, Prof. E. Dale Abel of the Department of Medicine at the David Geffen School of Medicine at UCLA stands out as the most prolific author. Prof. Abel is renowned for his extensive research on endocrine metabolic diseases, particularly focusing on the metabolic regulation of the cardiovascular system and mitochondrial biology. Future research trends will focus on the pathogenesis of mitochondrial dysfunction in the diabetic heart. This includes exploring specific molecular and cellular pathways that could potentially be targeted for therapeutic intervention. The emphasis will likely be on identifying and validating new therapeutic targets that address the underlying mechanisms contributing to mitochondrial impairment in DCM. This focus not only promises to enhance our understanding of the disease process but also to open new avenues for treatment strategies that could mitigate the progression of diabetic cardiomyopathy.

## Data Availability

The original contributions presented in the study are included in the article. Further inquiries can be directed to the corresponding author.
